# Identification and analysis of DNA methylation-driven signatures for prognostic and immune microenvironments evaluation in hepatocellular carcinoma

**DOI:** 10.3389/fgene.2022.1022078

**Published:** 2022-10-10

**Authors:** Bingbing Shen, Zhen Wen, Gang Lv, Jianguo Wang, Ruijie Han, Jianxin Jiang

**Affiliations:** ^1^ Department of Hepatobiliary Surgery, Renmin Hospital of Wuhan University, Wuhan, China; ^2^ Department of Anaesthesiology, Renmin Hospital of Wuhan University, Wuhan, China

**Keywords:** hepatocellular carcinoma, DNA methylation, immune, clinical, mutation, prognostic model, immunotherapy, bioinformatics

## Abstract

Liver cancer is the main reason of cancer deaths globally, with an unfavorable prognosis. DNA methylation is one of the epigenetic modifications and maintains the right adjustment of gene expression and steady gene silencing. We aim to explore the novel signatures for prognosis by using DNA methylation-driven genes. To acquire the DNA methylation-driven genes, we perform the difference analysis from the gene expression data and DNA methylation data in TCGA or GEO databases. And we obtain the 31 DNA methylation-driven genes. Subsequently, consensus clustering analysis was utilized to identify the molecular subtypes based on the 31 DNA methylation-driven genes. So, two molecular subtypes were identified to perform those analyses: Survival, immune cell infiltration, and tumor mutation. Results showed that two subtypes were clustered with distinct prognoses, tumor-infiltrating immune cell and tumor mutation burden. Furthermore, the 31 DNA methylation-driven genes were applied to perform the survival analysis to select the 14 survival-related genes. Immediately, a five methylation-driven genes risk model was built, and the patients were divided into high and low-risk groups. The model was established with TCGA as the training cohort and GSE14520 as the validation cohort. According to the risk model, we perform the systematical analysis, including survival, clinical feature, immune cell infiltration, somatic mutation status, underlying mechanisms, and drug sensitivity. Results showed that the high and low groups possessed statistical significance. In addition, the ROC curve was utilized to measure the accuracy of the risk model. AUCs at 1-year, 3-years, and 5-years were respectively 0.770, 0.698, 0.676 in training cohort and 0.717, 0.649, 0.621 in validation cohort. Nomogram was used to provide a better prediction for patients’ survival. Risk score increase the accuracy of survival prediction in HCC patients. In conclusion, this study developed a novel risk model of five methylation-driven genes based on the comprehensive bioinformatics analysis, which accurately predicts the survival of HCC patients and reflects the immune and mutation features of HCC. This study provides novel insights for immunotherapy of HCC patients and promotes medical progress.

## Introduction

In global cancer statistics, the incidence and mortality of hepatocellular carcinoma (HCC) rank top 10, which foster a considerable threat to life and health of human (Singal et al., 2020; Siegel et al., 2021; Sung et al., 2021). HCC is the most common style of liver cancer and accounts for more than 90% of all cases (Llovet et al., 2021). The treatment of HCC has noticeable improvements, including surgery and non-surgery therapy, but the treatment effect is still not ideal (Faivre et al., 2020; Petrowsky et al., 2020; Pillai et al., 2020; Rebouissou and Nault, 2020). Therefore, to improve the current status and bring benefits to the treatment of HCC patients, we must explore new molecular mechanisms of HCC progression.

DNA methylation is one of the epigenetic modifications and maintains correct adjustment of gene expression and steady gene silencing (Kulis and Esteller, 2010). Total DNA hypomethylation plays a vital role in Tumorigenesis, which is associated with chromosomal instability (Kawano et al., 2014).

We selected shared genes and DEGs based on the expression data of TCGA and GSE14520. Next, based on the expression and DNA methylation data of TCGA, we identified the methylation-driven genes. The consensus clustering analysis was employed to establish molecular subtypes by taking advantage of methylation-driven genes. Based on the molecular subtypes, we perform the following analysis: survival analysis, immune cell infiltration analysis, and mutation analysis.

Furthermore, in order to construct a prognostic model with as few genes as possible and high accuracy, the methylation-driven genes were employed to carry out the survival analysis to select the survival-related genes. Immediately, a five methylation-driven genes risk model was built, with TCGA as the training cohort and GSE14520 as the validation cohort. From the risk model, we perform the systematical analysis, including survival, clinical feature, immune cell infiltration, somatic mutation status, underlying mechanisms, and drug sensitivity. ROC curve was utilized to assess the accuracy of risk model. Nomogram was used to provide a better prediction for patients’ survival. Finally, our study established and validated a predictive model for HCC based on the methylation-driven genes, which was utilized to predict the prognosis of HCC patients effectively.

**FIGURE 1:**
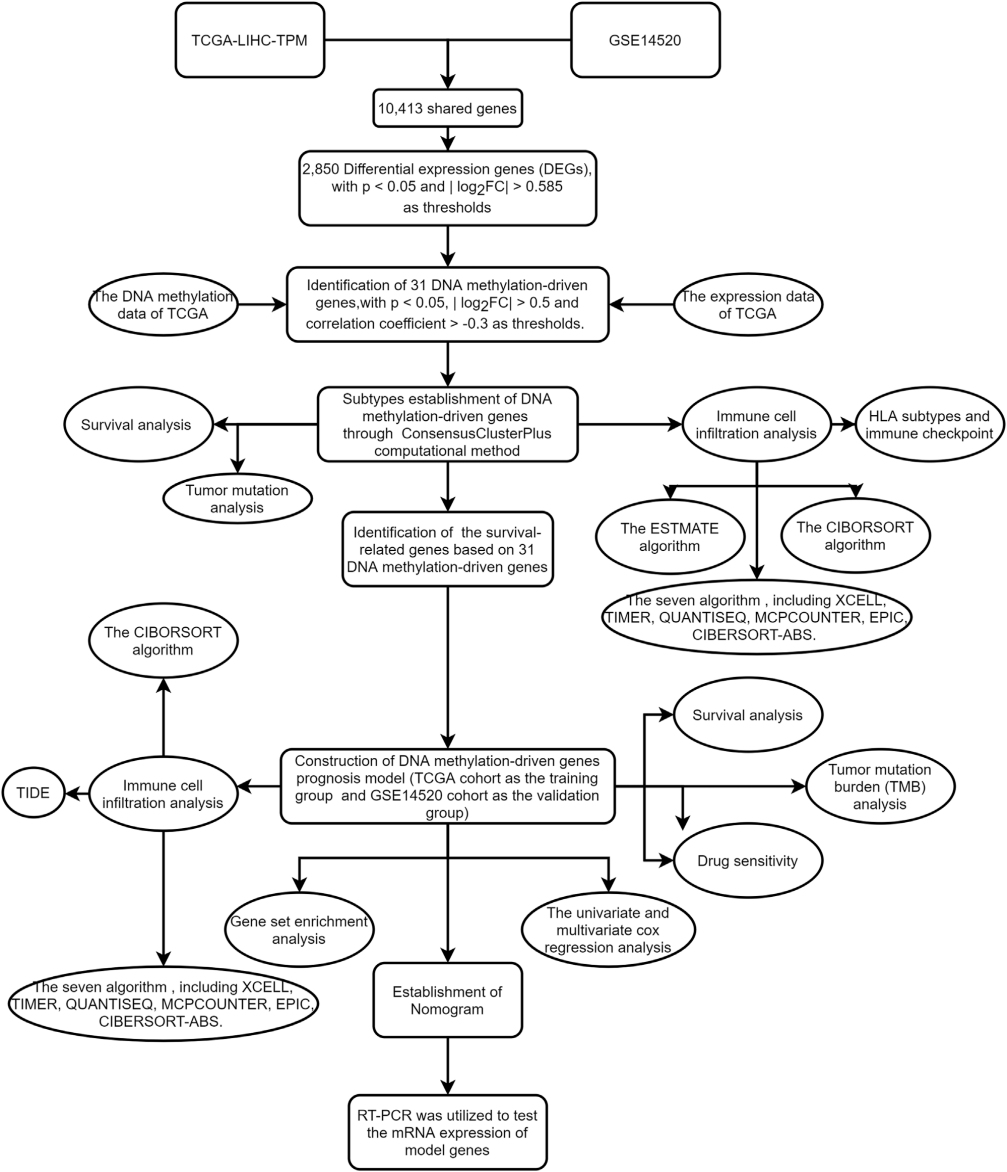
The workflow of the study.

Although most studies were based on bioinformatics analysis, it may contribute to immunotherapy of HCC and promote scientific advances in the future. The workflow of the study is shown in Figure 1.

## Materials and methods

### Data collection

Gene expression and clinical files were downloaded from GEO (https://www.ncbi.nlm.nih.gov/geo/) and TCGA databases (https://portal.gdc.cancer.gov/repository). DNA methylation and copy number variation (CNV) files were also obtained from TCGA (Wang et al., 2016). The gene expression and clinical files of GSE14520 (Chen et al., 2021) originated from the GEO database and are based on the GPL3921 platform [HT_HG-U133A] (Affymetrix HT Human Genome U133A Array), covering 241 normal and 247 tumor samples. The mRNA expression of TCGA (TPM format) includes 50 normal and 374 tumor samples. DNA methylation data of TCGA have 50 normal and 379 tumor samples. The “sva” R package was applied for data correction and normalization.

### Acquisition of 31 DNA methylation-driven genes

The “limma” package of R language was applied to identify differentially expressed genes (DEGs) in TCGA database, with *p* < 0.05 and | log_2_FC| > 0.585 as thresholds. And 2850 DEGs were selected. The “MethylMix” package of R language was employed to select the methylation-driven genes in TCGA, with *p* < 0.05, | log_2_FC| > 0.5 and correlation coefficient > −0.3 as thresholds. Finally, 31 methylation-driven genes were identified. Those genes were showed in Supplementary Table S1.

### Consensus clustering

The expression data of 31 methylation-driven genes were extracted from TCGA cohort. The “ConsensusClusterPlus” R package was utilized to cluster samples (Wilkerson and Hayes, 2010). Clustering analysis was overlapped 50 times to enhance the availability of Clustering.

### Construction of DNA methylation-driven genes prognosis model

Firstly, univariate Cox and survival analyses were applied to acquire the prognostic DNA methylation-driven genes. Next, the TCGA cohort was regarded as a training group, and the GSE14520 cohort was followed as a testing group. According to the prognostic DNA methylation-driven genes, Lasso-Cox regression analysis was applied to select genes to minimize the overfitting risk and construct a risk prediction model. The risk score was calculated through the following formula:

Risk score=∑[Exp(Gene) ∗ coef(Gene)]

Exp (Gene) is the expression of DNA methylation-driven genes, and coef (Gene) is the associated regression coefficient.

Survival curves were designed using the “survival” and “survminer” R packages. ROC (receiver operating characteristic) curves were created by using the “survival”, “survminer” and “timeROC” R packages.

### Tumor mutation burden

Cluster group and risk score were utilized to classify the raw mutation annotation format (MAF). The TMB score for each patient was determined based on the somatic mutation data. The “maftools” R package was applied to perform mutation analysis and draw the waterfall. The “survival” and “survminer” R packages were used to analyze and design the survival curve.

### Tumor immunity analysis

The ESTIMATE algorithm (Yoshihara et al., 2013) was applied to calculate stromal, immune, and estimate scores. The following R packages were used to acquire and visualize results of ESTIMATE algorithm based on TCGA database, including “limma”, “estimate” and “ggpubr” packages. The CIBERSORT algorithm (Newman et al., 2015) was used to evaluate the correlation between 22 immune cells and different groups. In addition, based on the TCGA-LIHC-TPM cohort, seven tools were utilized to predict infiltration, including XCELL, TIMER, QUANTISEQ, MCPCOUNTER, EPIC, and CIBERSORT-ABS. The following R packages were used: “limma”, “scales”, “ggplot2″, “ggtext”, “reshape2″, “tidyverse” and “ggpubr".

### GSEA

The GSEA algorithm (Subramanian et al., 2005) (Mootha et al., 2003) is an abundance way to calculate the measured proportion of specific paths or features in different clusters. The “limma”, “org.Hs.eg.db”, “clusterProfiler” and “enrichplot” R packages were applied to perform GSEA analysis. Gene sets (c5.go.symbols.gmt and c2.cp.kegg.symbols.gmt) were used to perform GSEA analysis, where *p* < 0.05 and FDR <0.05. And the top 5 were shown.

### Nomogram and calibration

R language was used to build a predictive Nomogram. The following packages were used, including “survival”, “regplot” and “rms”. The calibration curve was applied to quantify the consistency between predictive and actual results for 1-, 3-, and 5-year survival time.

### Survival analysis and clinical correlation analysis

The correlation between expression of 5 methylation-driven genes and survival time was visualized through drawing a survival curve based on R language. In addition, R language also was applied to visualize the correlation between methylation level of 5 methylation-driven genes and survival time. The “survival” and “survminer” R packages were employed to paint survival curve. The “ComplexHeatmap” package was used to draw a clinical correlation heatmap for high-risk and low-risk groups.

### TIDE

TIDE (http://tide.dfci.harvard.edu/) may evaluate multiple published transcriptomic biomarkers to predict patient response according to tumor pre-treatment expression profiles. Our study used TIDE to explore the relation between TIDE score and high-risk and low-risk groups.

### Cell culture

HCC cell lines (MHCC-97H, Hep-G2) and normal liver cell line (MIHA) were purchased from the Cell Bank of Chinese Academy of Science (Shanghai, China). Subsequently, all cell lines were cultivated into DMEM (Gibco) with 10% FBS (Gibco) and stored in 5% CO_2_ at 37°C.

### RT-PCR

Firstly, according to the manufacturer’s instructions, we performed the extraction of total RNA. Secondly, we acquired the cDNA through reverse transcription. Finally, we carried out the RT-PCR. The primers were shown in the Supplementary Material S1.

### Drug sensitivity analysis

The IC50 value, collected from the GDSC website (https://www.cancerrxgene.org/), was calculated to speculate the available compounds for treating HCC patients. The “oncoPredict” package (Maeser et al., 2021) was utilized to observe the therapeutic effect of medications on high-risk and low-risk groups. The criterion is *p* < 0.001.

### Statistical analysis

R language (version:4.2.1) was employed to carry out statistical analysis. Data analysis was deemed as meaningful data, while the formula *p* < 0.05 showed the meaning of a statistically significant difference.

## Results

### Workflow of research

The flowchart of this study is shown in Figure 1. The precise procedure is as follows: First, we obtained 31 DNA methylation-driven genes through acquiring the shared genes between GSE14520 and TCGA data, performing the difference analysis. Second, ConsensusClusterPlus computational method was utilized to classified the HCC patients into two subtypes based on the 31 DNA methylation-driven genes. Finally, we built a novel DNA methylation prognosis model.

### Identification of DNA methylation-driven genes

**FIGURE 2:**
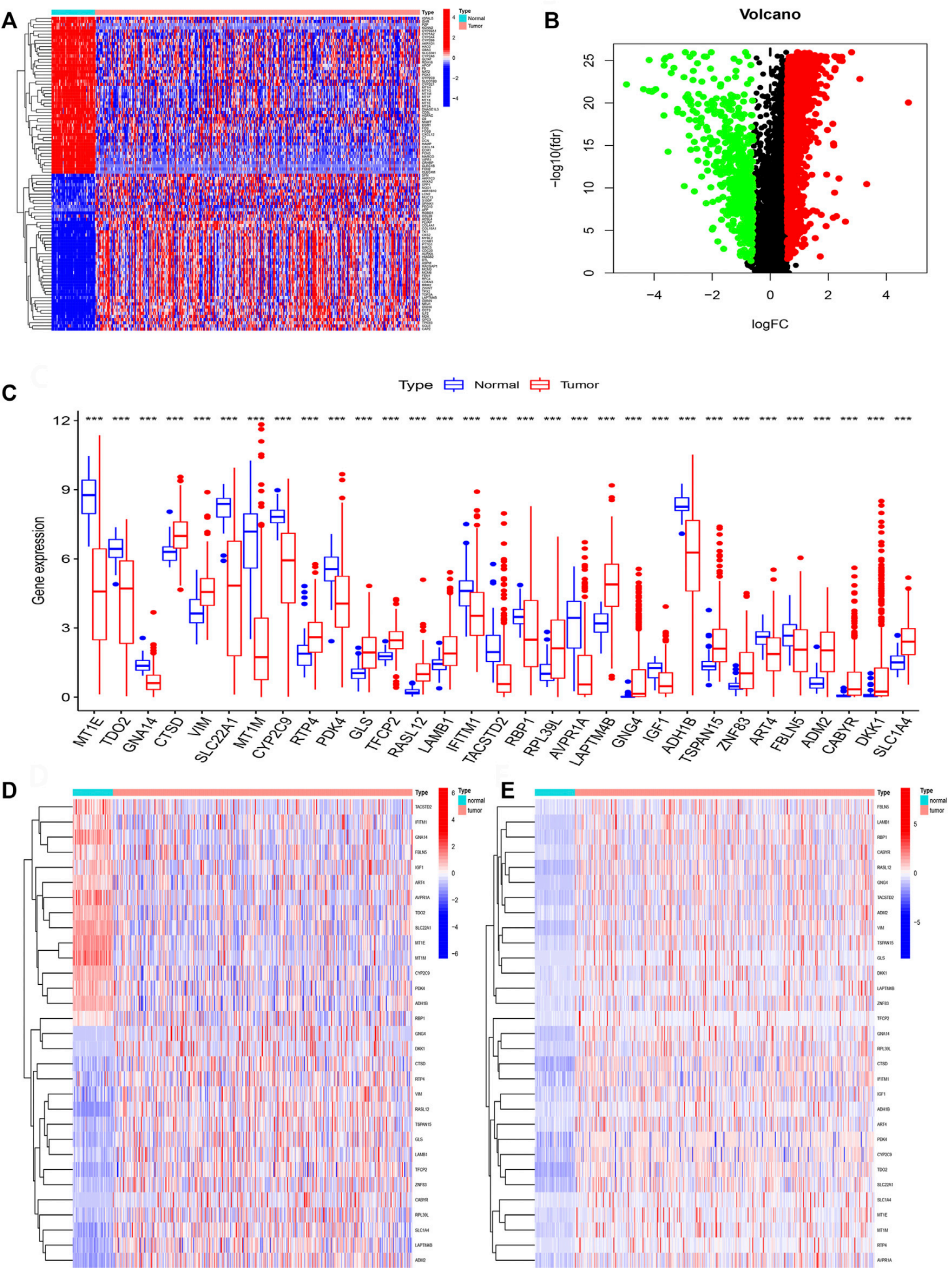
Identification of DNA methylation-driven genes. **(A)** The heatmap of DEGs. **(B)** The volcano map of DEGs. **(C)** Expression of 31 DNA methylation-driven genes. **(D)** The heatmap of methylation levels about 31 DNA methylation-driven genes. **(E)** The heatmap of expression of 31 DNA methylation-driven genes.

We firstly acquired the 10,413 shared genes between TCGA and GSE14520. Next, we obtained 2850 DEGs through performing the difference analysis based on TCGA database. DEGs (Differential expression gene) heatmap was shown in Figure 2A, and volcano map was shown in Figure 2B. Moreover, the DNA methylation and expression data of TCGA were used to acquire the DNA methylation-driven genes, and we acquired the 31 methylation-driven genes (Supplementary Table S1). The expression of 31 methylation-driven genes was tested using the TCGA database (Figure 2C). The heatmap of methylation level (Figure 2D) and heatmap of gene expression (Figure 2E) showed that expression level and methylation level of 31 DNA methylation-driven genes in each sample. And we found that most tumor samples have relatively high methylation levels.

### Subtypes establishment of DNA methylation-driven genes

**FIGURE 3:**
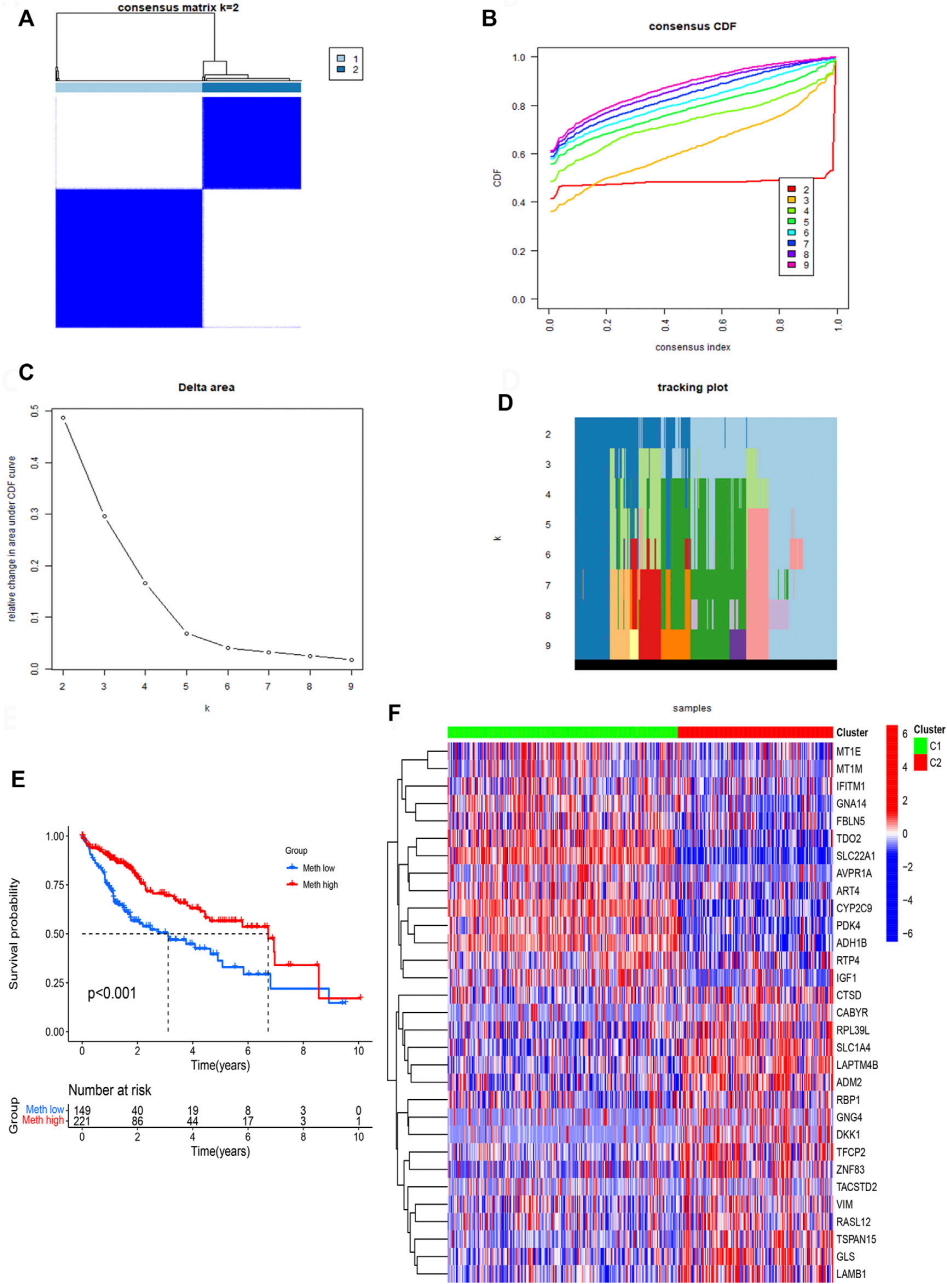
Identification of two subtypes based on Consensus clustering analysis. **(A)** Consensus clustering at *k* = 2. **(B)** Empirical CDF plots corresponding to each k. **(C)** Delta area diagram at different k. **(D)** Item tracking plot at each k. **(E)** Survival analysis of HCC patients in two subtypes. **(F)** The heatmap of 31 DNA methylation-driven genes in two subtypes.

ConsensusClusterPlus computational method was used to divide the HCC patients into two subtypes according to the expression file of 31 DNA methylation-driven genes (Figure 3A). CDF (Cumulative distribution function) was applied to obtain the k value while the distribution reached a maximum means applicable stability (Figure 3B). The Delta region graph uncovered that the region under the curve slowly climbed while k = 2 (Figure 3C). Furthermore, the tracking graph unfolded the consensus clustering of projects at different k values (Figures 3D,E). The survival curve uncovered that Cluster 2 had poor survival time, and Cluster1 had a better survival advantage. In addition, the heatmap (Figure 3F) showed the expression of 31 DNA methylation-driven genes in two subtypes.

### Cluster subtypes are associated with tumor microenvironment features

**FIGURE 4:**
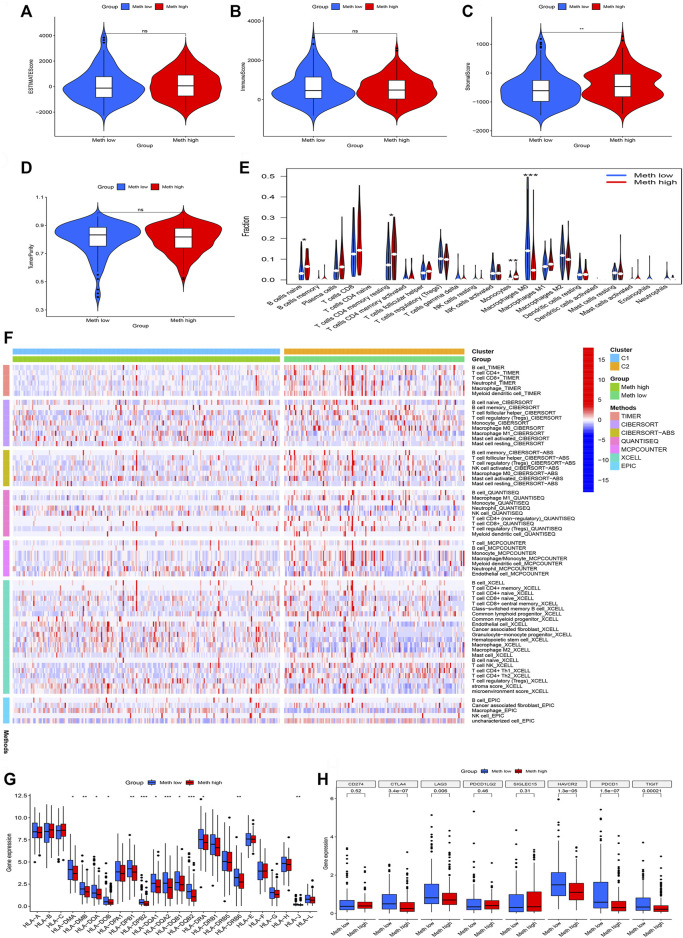
Immune cell infiltration in two subtypes. **(A)** ESTIMATE Score in two subtypes. **(B)** Immune Score in two subtypes. **(C)** Stromal Score in two subtypes. **(D)** TumorPurity Score in two subtypes. **(E)** The violin map based on the CIBERSORT in two subtypes. **(F)** The heatmap of seven immune methods in two subtypes. **(G)** The difference between HLA subtypes in two subtypes. **(H)** The difference between immune checkpoints in two subtypes.

The ESTIMATE algorithm was used to assess the immune cell composition in each sample. Tumor microenvironment difference of two Subtypes was identified through ESTIMATE score, stromal score, Immune score, and tumor purity. We found that Meth-high group had a higher stromal score (Figure 4C). However, the difference does not exist in ESTIMATE score (Figure 4A), Immune score (Figure 4B), and tumor purity (Figure 4D). Next, the CIBORSORT algorithm was utilized to analyze the immune cell difference between two Subtypes. The followed immune cells were identified, naïve B cells, resting memory CD4 T-cells, monocytes, and M0 macrophages (Figure 4E). To further observe the immune cell infiltration of two DNA methylation-driven Subtypes, we applied seven algorithms to draw a heatmap of immune cell infiltration. We deem that the Meth-low group had a higher immune infiltration level (Figure 4F). We then analyzed the correlation between two Subtypes and expression of HLA subtypes as well as immune checkpoints. We found that most HLA subtypes were highly expressed in Meth-low group (Figure 4G). For Example, *HLA-DMA*, *HLA-DMB*, *HLA-DOA*, and *HLA-DOB*. We also observed that most immune checkpoints were highly expressed in Meth-low group (Figure 4H), such as *CTLA4*, *LAG3*, *HAVCR2*, *PDCD1*, and *TIGIT*. Therefore, we deem that the Cluster 2 or Meth-low group may benefit from the immune checkpoint inhibitor therapy in our subtypes.

### Cluster subtypes are associated with tumor mutation features

**FIGURE 5:**
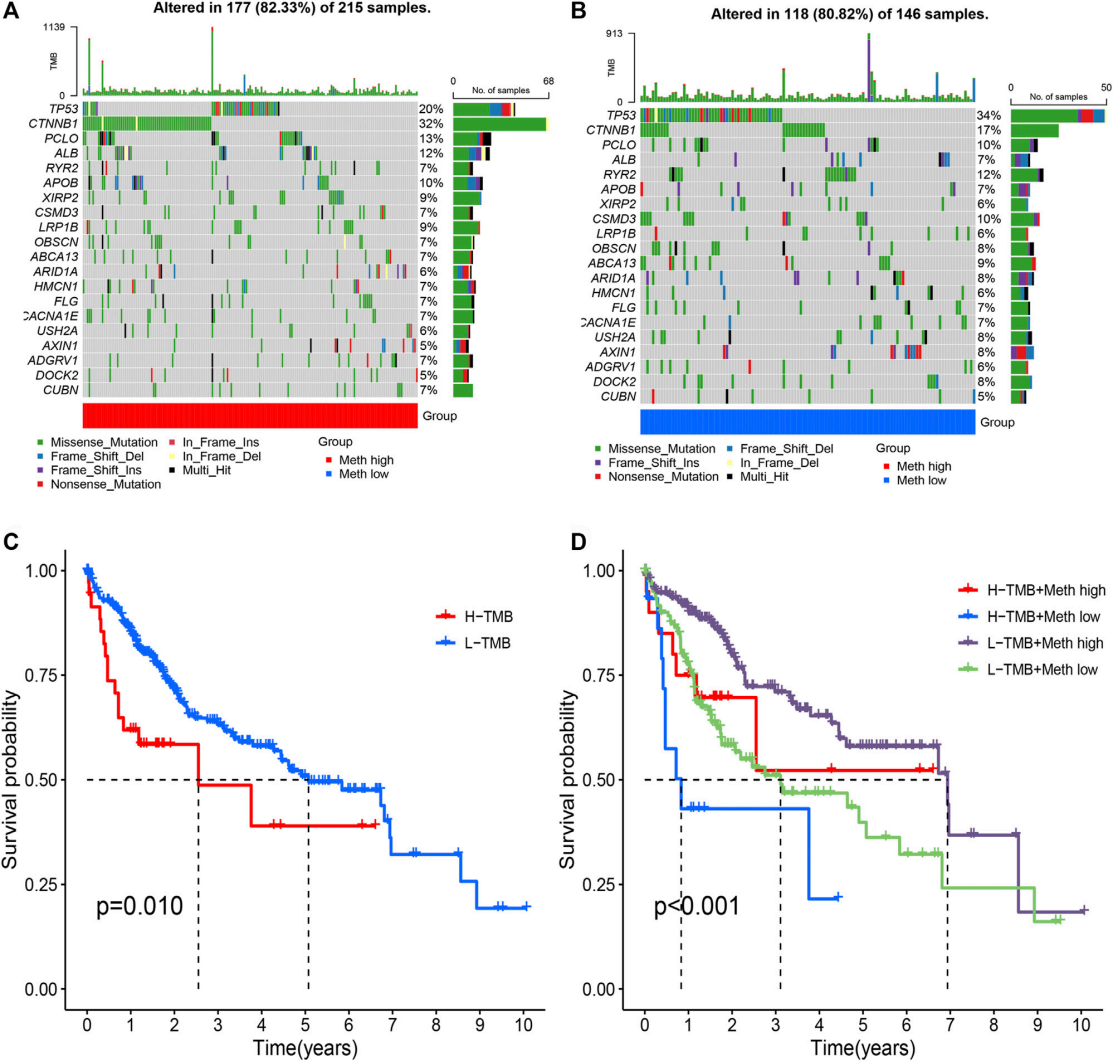
Tumor mutation analysis in two subtypes. **(A)** The mutation waterfall map of Cluster 1 group (Meth high). **(B)** The mutation waterfall map of Cluster 2 group (Meth low). **(C)** Survival analysis of two groups. **(D)** Survival analysis of four groups.

From the waterfall plot (Figures 5A,B), we found that TMB differences exist in two subtypes. We observed 177 (82.33%) of 215 samples in Meth-high group (Figure 5A) and 118 (80.82%) of 146 samples in Meth-low group (Figure 5B). In addition, we also observed that *CTNNB1*, *PCOL*, *APOB*, *XIRP2*, *LRP1B*, *HMCN1*, *ADGRV1*, and *CUBN* have a higher TMB in Meth-high group, but *TP53*, *RYR2*, *CSMD3*, *OBSCN*, *ABCA13*, *ARID1A*, *USH2A*, *AXIN1*, *DOCK2* have a higher TMB in Meth-low group. And from the survival curve (Figure 5C), we deem that the H-TMB group possesses a poor survival time. In addition, we also found that High-TMB and Low-methylation groups are closely associated with worst prognosis, low-TMB and high-methylation groups are closely associated with best prognosis (Figure 5D).

### Construction of DNA methylation-driven genes prognosis model

**FIGURE 6:**
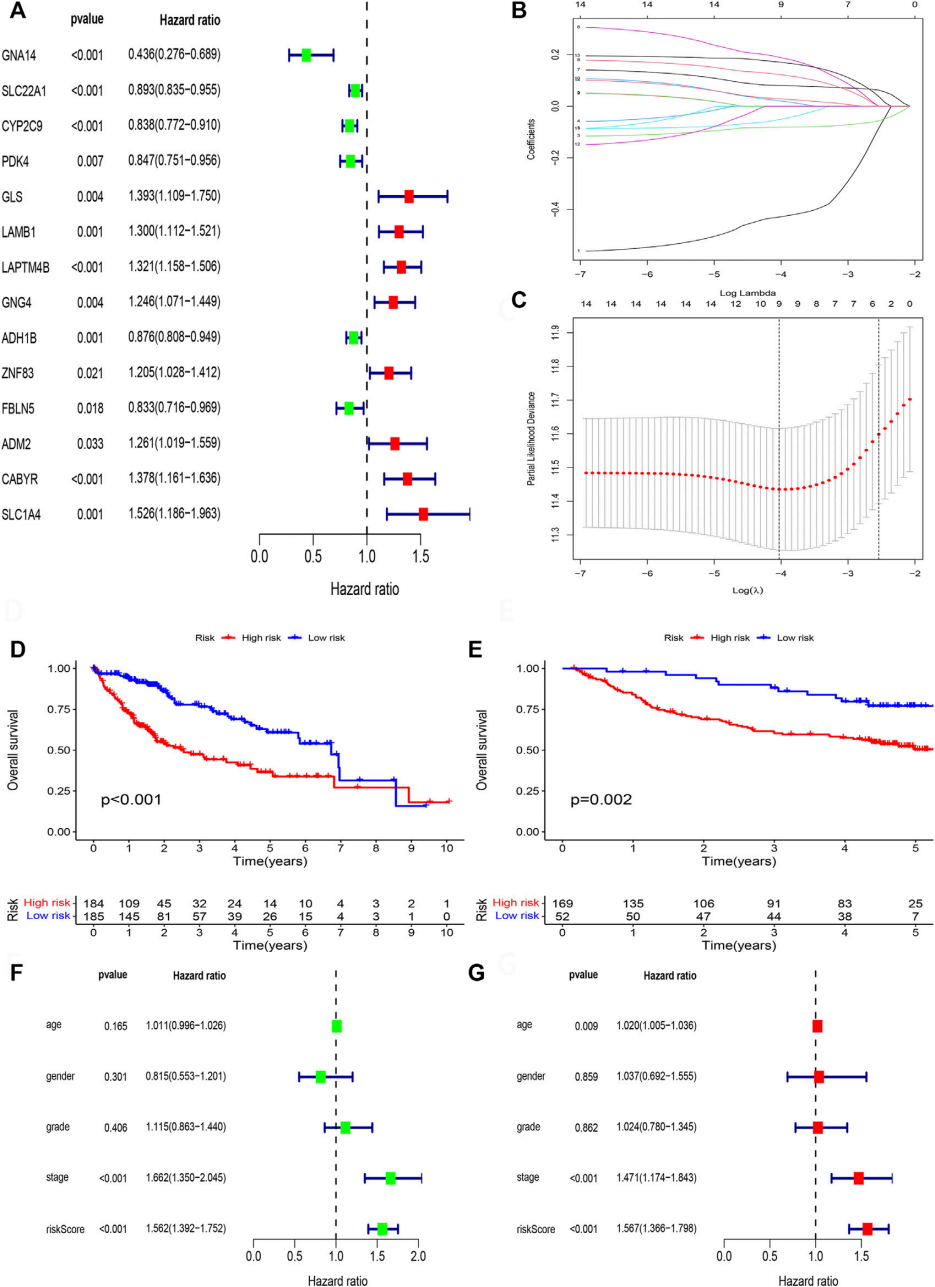
Lasso regression analysis. **(A)** The univariate cox regression analysis of 31 DNA methylation-driven genes. **(B)** LASSO coefficient distribution of DNA methylation-driven genes. **(C)** The tuning parameter (*λ*) in the LASSO model. **(D)** Survival curve of the two risk groups derived from the five-gene signature in the TCGA cohort. **(E)** Survival curve of the two risk groups derived from the five-gene signature in the GSE14520 cohort. **(F)** Univariate Cox regression analysis of risk scores and clinical features. **(G)** Multivariate Cox regression analysis of risk scores and clinical features.

We perform survival filtering based on the above 31 methylation-driven genes to select survival-related genes. Thus, we obtain 14 survival-related genes (Figure 6A). There are *GNA14*, *SLC22A1*, *CYP2C9*, *PDK4, GLS*, *LAMB1*, *LAPTM4B*, *GNG4*, *ADH1B*, *ZNF83*, *FBLN5*, *ADM2*, *CABYR*, and *SLC1A4*. Moreover, LASSO regression analysis was applied to construct the methylation-driven genes prognosis model. Cross-validation was applied to acquire the best value to identify further the prognosis-associated genes (Figures 6B,C). Finally, five methylation-related genes were identified to build a risk model, including *GNA14*, *CYP2C9*, *LAMB1*, *GNG4*, and *CABYR*. The risk score was calculated by this formula: expression of *GNA14* * (−0.756524222481111) + expression of *CYP2C9* * (−0.0995480923807365) + expression of *LAMB1* * (0.265282085430652) + expression of *GNG4* * (0.157119364590744) + expression of *CABYR* * (0.237082502752391).

Next, patient samples, including training group (TCGA cohort) and validation group (GSE14520 cohort), were divided into high-risk group and low-risk group based on the median score of risk value. From survival curve, we deem that OS is shorter in the high-risk than low-risk group in TCGA cohort (Figure 6D) and GSE14520 cohort (Figure 6E). Based on the univariate (Figure 6F) and multivariate cox regression analysis (Figure 6G) in TCGA cohort, we deem that risk score and stage were independent prognosis factors of HCC patients.

**FIGURE 7:**
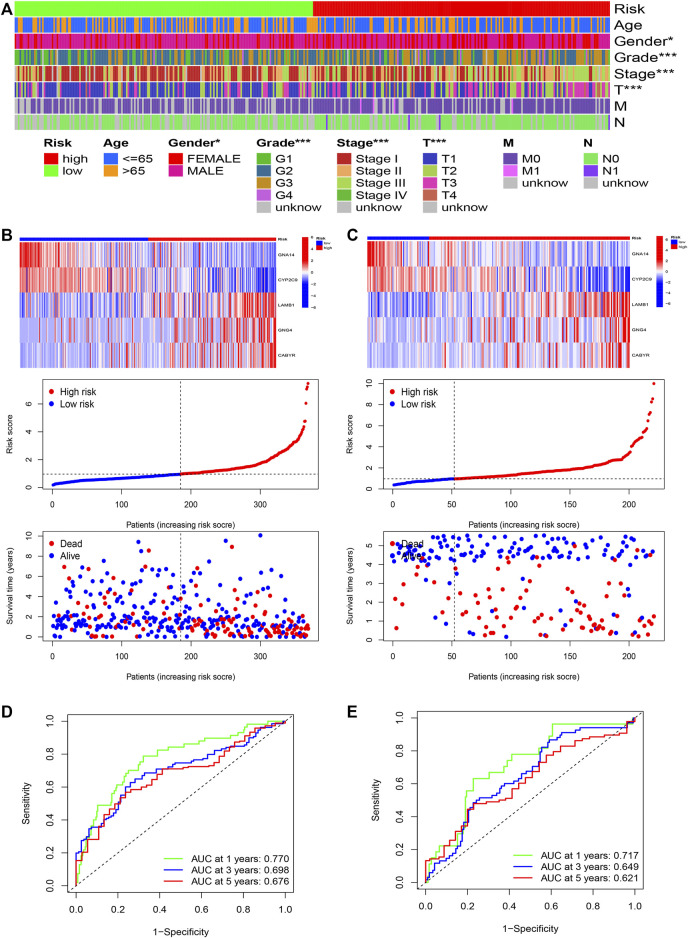
The risk model of five-gene signature. **(A)** The correlated heatmap of the risk score and clinical features. (*p < 0.05; **p < 0.01; ***p < 0.001). **(B)** The five-gene mRNA expression, risk score distribution, and the related survival data in the TCGA cohort. **(C)** The five-gene mRNA expression, risk score distribution, and the related survival data in the GSE14520 cohort. **(D)** ROC curves for 1-, 3-, and 5-years overall survival predictions in the TCGA cohort. **(E)** ROC curves for 1-, 3-, and 5-years overall survival predictions in the GSE14520 cohort.

Furthermore, R language was utilized to draw the heatmap of risk value and clinical features. And from the heatmap found that the risk value is closely associated with gender, grade stage, TNM stage, and T stage (Figure 7A). Risk heatmap, risk score distribution, and survival status distribution were painted, respectively (Figures 7B,C). ROC curve was generated in the TCGA (Figure 7D) and GSE14520 (Figure 7E) cohorts, and AUCs at 1 year, 3-years, and 5 years were respectively 0.770, 0.698, 0.676 in training cohort and 0.717, 0.649, 0.621 in validation cohort. Results showed excellent predictive ability in TCGA and GSE14520 cohorts.

### Correlation between risk model and immune features

**FIGURE 8:**
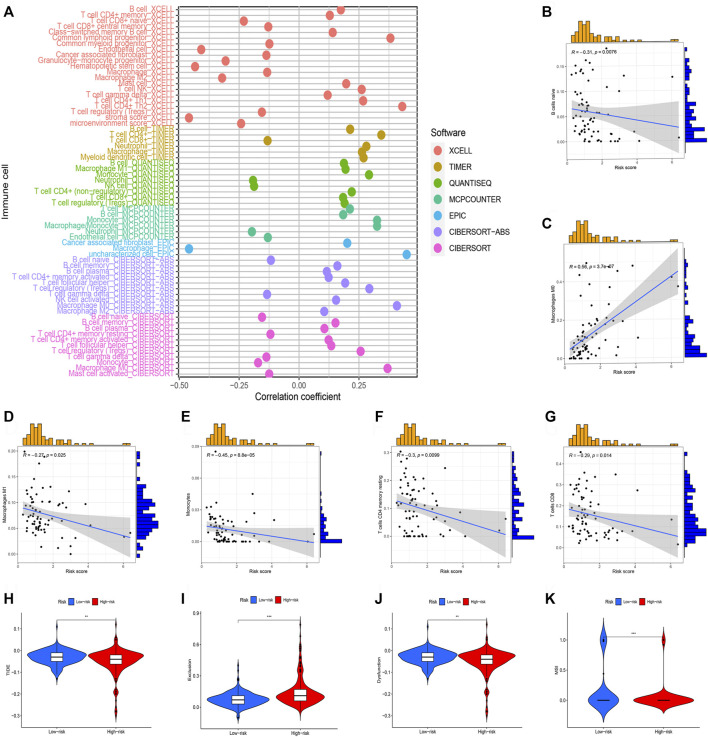
Immune cell infiltration analysis of different risk groups. (*p < 0.05; **p < 0.01; ***p < 0.001). **(A)** Correlation between risk scores and immune cells. **(B)** The correlation between risk score Naive B cells. **(C)** The correlation between risk score M0 macrophages. **(D)** The correlation between risk score M1 macrophages. **(E)** The correlation between risk score Monocytes. **(F)** The correlation between risk score resting memory CD4 T-cells. **(G)** The correlation between risk score CD8 T-cells. **(H)** TIDE score in different risk groups. **(I)** Exclusion score in different risk groups. **(J)** Dysfunction score in different risk groups. **(K)** MSI score between different risk groups.

Subsequently, we perform the immune cell infiltration analysis. Firstly, the bubble graph (Figure 8A) showed the relationship between the risk score and various immune cells based on the seven methods, including CXELL, TIMER, QUANTISEQ, MCPCOUNTER, EPIC, and CIBORSORT-ABS. We uncovered that risk score is closely associated with immune cell infiltration. Secondly, the CIBERSORT algorithm was utilized to assess the relationship between risk score and immune, and we only showed significant results. Results uncovered that M0 macrophages is positive related to risk score (Figure 8C), but naïve B cells (Figure 8B), M1 macrophages (Figure 8D), monocytes (Figure 8E), resting memory CD4 T-cells (Figure 8F), CD8 T-cells (Figure 8G) were negatively related to risk score. Lastly, TIDE was used to evaluate the clinical efficacy of immunotherapy in HCC patients. Results showed that high-risk groups possess a lower TIDE level than low-risk groups (Figures 8H–K). Therefore, we deem that HCC patients in high-risk group were more likely to benefit from immunotherapy.

### Correlation between risk model and tumor mutation features

**FIGURE 9:**
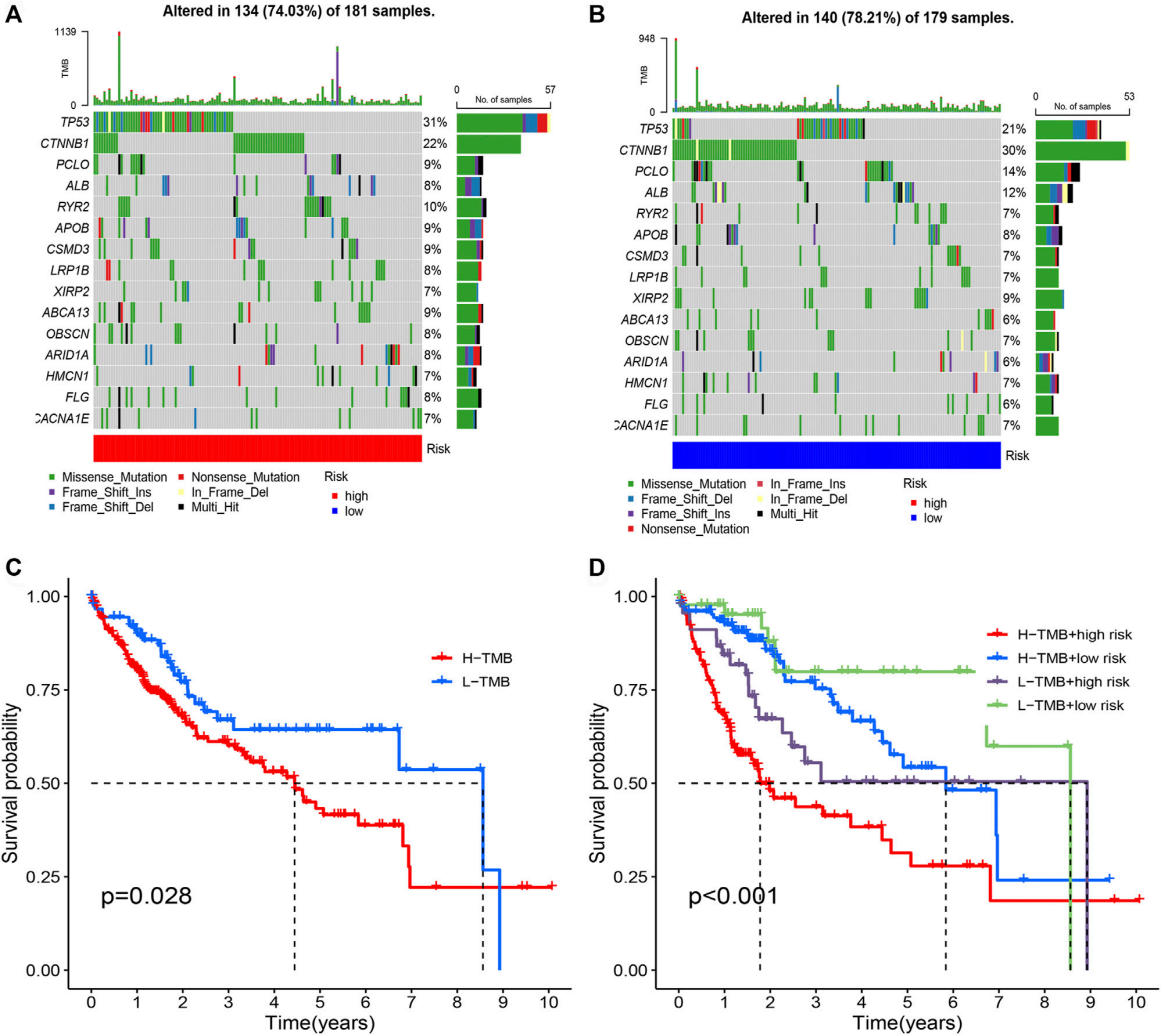
Tumor mutation analysis in different risk groups. **(A)** The mutation waterfall map of high-risk group. **(B)** The mutation waterfall map of high-risk group. **(C)** Survival analysis of two groups. **(D)** Survival analysis of four groups.

Fhrthermore, we focus on the tumor somatic mutation in the prognosis model based on the TCGA cohort. From the waterfall graph of TMB, we found that mutation difference exists in risk groups (Figures 9A,B). The most apparent somatic mutations in TP53 and CTNNB1 were in the two groups. However, we discovered that the mutation rate of TP53 accounts for 21% in the high-risk group but 34% in the low-risk group. We also observed that the mutation rate of CTTNB1 accounts for 22% in the high-risk group but 30% in the low-risk group. Thus, we deemed that the somatic mutation of TP53 and CTTNB1are the most obvious between two groups. Thus, we deemed that the risk group of risk model may bring us novel insights for Tumorigenesis. Subsequently, we perform the survival analysis based on the risk group. Results showed that the high-mutation group possesses a shorter OS than the low-mutation group (Figure 9C). In addition, we also found that high-mutation and high-risk groups possess a shorter OS than other groups (Figure 9D). Therefore, we concluded that low-mutation rate and low-risk was a protective factor for HCC patients.

### GSEA

**FIGURE 10:**
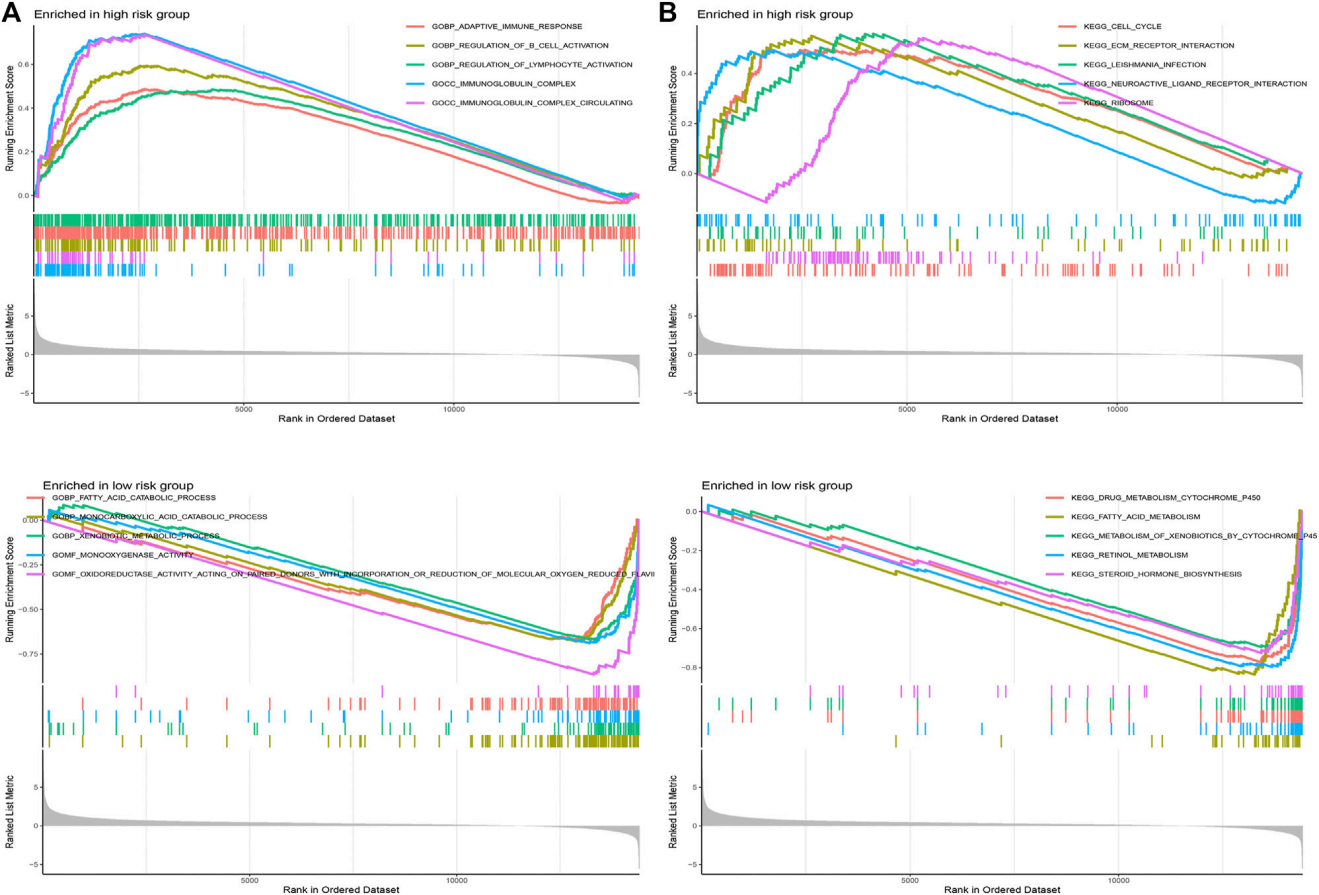
Gene set enrichment analysis. **(A)** GO analysis in different risk groups. **(B)** KEGG analysis in different risk groups.

GSEA was applied to explore the function enrichment and KEGG pathway based on the high and low-risk groups in the TCGA cohort. From the GO analysis (Figure 10A), we found adaptive immune response, regulation of B cell activation, regulation of lymphocyte activation, immunoglobulin complex, and immunoglobulin complex circulating in the high-risk group. Fatty acid catabolic process, monocarboxylic acid catabolic process, xenobiotic metabolic process, monooxygenase activity, and oxidoreductase activity acting on paired donors with incorporation or reduction of molecular oxygen reduced flavoprotein as one donor and incorporation of one atom of oxygen in the low-risk group. From KEGG analysis (Figure 10B), cell cycle, ECM receptor interaction, leishmania infection, neuroactive ligand-receptor interaction, and ribosome in the high-risk group. Drug metabolism cytochrome p450, fatty acid metabolism, metabolism of xenobiotics by cytochrome p450, retinol metabolism, and steroid hormone biosynthesis in the low-risk group. Results showed that the majority of enrichment in the high-risk group is closely associated with the immune and the majority of enrichment in the low-risk group is closely associated with metabolism.

### Establishment of nomogram

**FIGURE 11:**
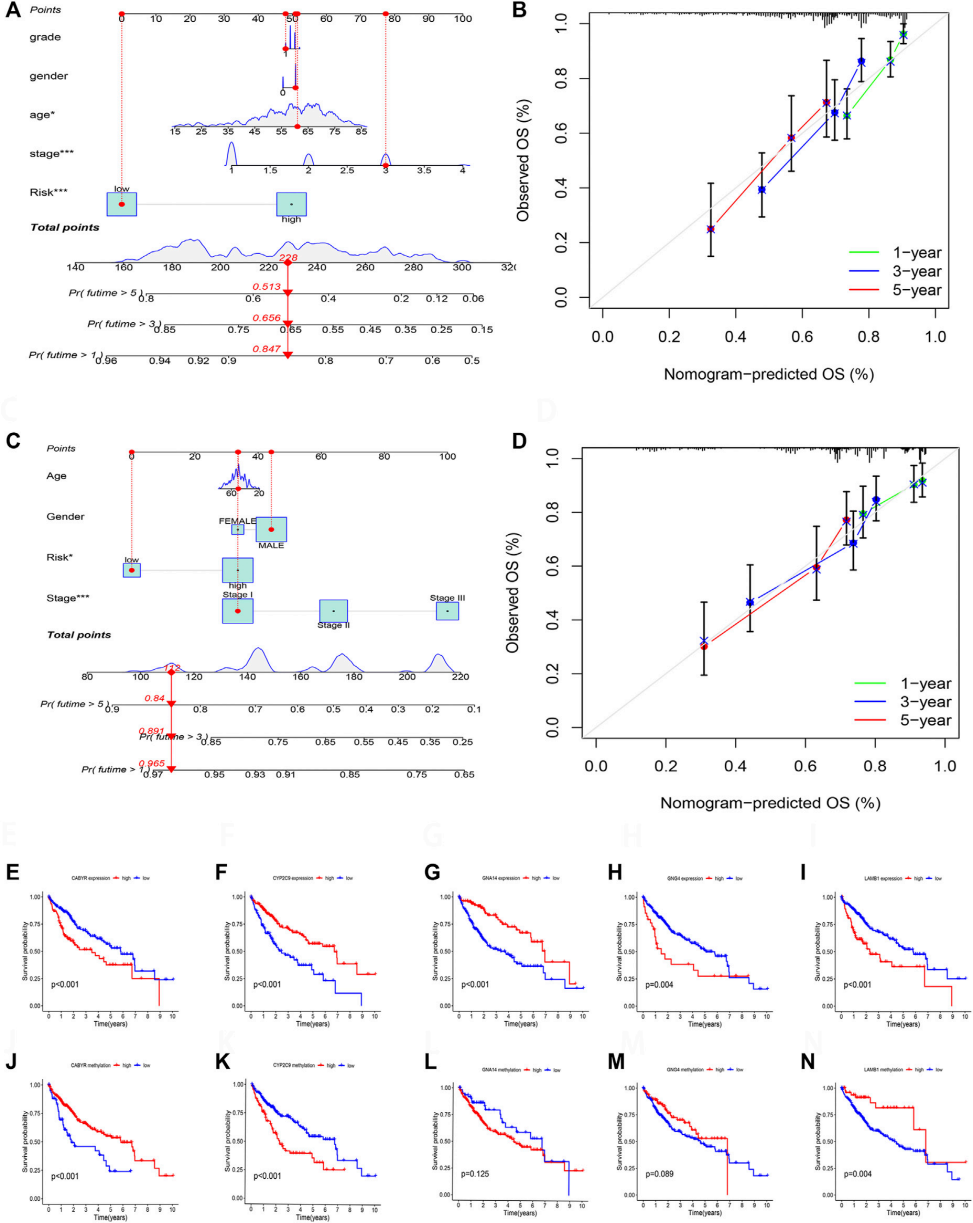
**(A)** The nomogram integrated the risk score and clinical features to predict the survival rate of the 1, 3, and 5 years in TCGA cohort. **(B)** The 1, 3, and 5 years OS calibration curves in TCGA cohort. **(C)** The nomogram integrated the risk score and clinical features to predict the survival rate of the 1, 3, and 5 years in GSE14520 cohort. **(D)** The 1, 3, and 5 years OS calibration curves in GSE14520 cohort. **(E)** Survival analysis of CABYR expression. **(F)** Survival analysis of CYP2C9 expression. **(G)** Survival analysis of GNA14 expression. **(H)** Survival analysis of GNG4 expression. **(I)** Survival analysis of LAMB1 expression. **(J)** Survival analysis of CABYR methylation level. **(K)** Survival analysis of CYP2C9 methylation level. **(L)** Survival analysis of GNA14 methylation level. **(M)** Survival analysis of GNG4 methylation level. **(N)** Survival analysis of LAMB1 methylation level.

According to the TCGA and GSE14520 cohorts, we built a Nomogram through integrating risk score and clinical features to strengthen further the predictive ability of HCC patients (Figures 11A,C). In addition, calibration graph of 1, 3, and 5- years OS uncovered that consistency exists in Nomogram prediction and actual observations (Figures 11B,D).

### Expression and survival analysis of 5 DNA methylation-driven genes

RT-PCR was performed to test the mRNA expression of 5 DNA methylation-driven genes. Results showed that the expression of *CYBYR*, *GNG4*, and *LAMB1* is higher in HCC cell lines than in normal liver cell line, and the expression of *CYP2C9* and *GNA14* is lower in HCC cell lines than in normal liver cell line (Supplementary Figure S1A). Next, survival analysis was utilized to assess the correlation between five genes of the prognosis model and survival time based on the expression and methylation level in TCGA cohort. Results revealed that high expression of *CYBYR* (*p* < 0.001), *GNG4* (*p* = 0.004), and *LAMB1* (*p* < 0.001) is closely associated with short OS, low expression of *CYP2C9* (*p* < 0.001), and *GNA14* (*p* < 0.001) is closely related to short OS (Figures 11E–I). Meanwhile, survival curve also revealed that the low-methylation level of *CABYR* (*p* < 0.001) and *LAMB1* (*p* = 0.004) is related to shorter OS, and the high-methylation story of *CYP2C9* (*p* < 0.001) is connected to shorter OS. However, the methylation level of *GNA14* (*p* = 0.125) and *GNG4* (*p* = 0.089) was not significantly correlated with survival (Figures 11J–N).

### Drug sensitivity

**FIGURE 12:**
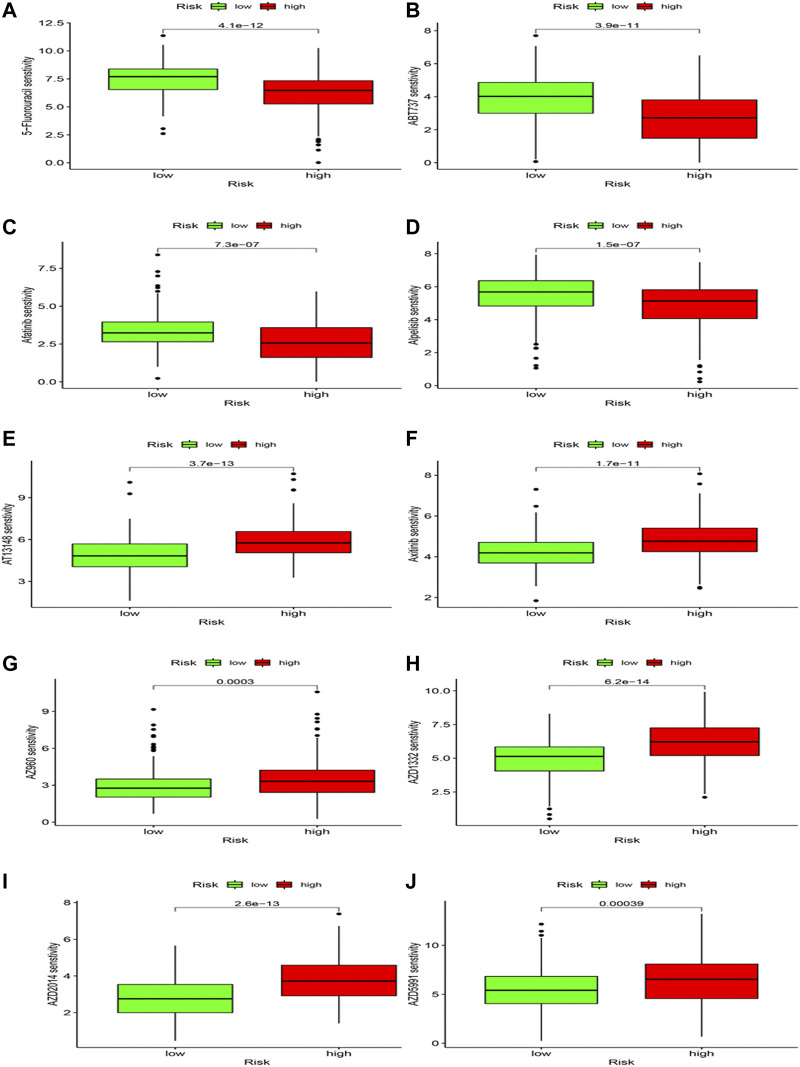
Drug sensitivity analysis.

Differences in drug sensitivity and risk group were analyzed to speculate the clinical application of risk model. Results showed that 103 drugs have good effects on HCC patients based on different risk groups (Supplementary Table S2; Figure 12), including 5-Fluorouracil, ABT737, Afatinib, and Alpelisib, AT13148, Axitinib, AZ960, etc. We concluded that 5-Fluorouracil (Figure 12A), ABT737 (Figure 12B), Afatinib (Figure 12C) and Alpoelisib (Figure 12D) possessed lower IC50 values compared to the high-risk group. Still, AT13148 (Figure 12E), Axitinib (Figure 12F), AZ960 (Figure 12G), AZD1332 (Figure 12H), AZD2014 (Figure 12I), and AZD5991 (Figure 12J) possessed lower IC50 values in the low-risk group, representing 5-Fluorouracil, ABT737, Afatinib and Alpoelisib were more effective in the high-risk group; AT13148, Axitinib, AZ960, AZD1332, AZD2014, and AZD5991 were more effective in the low-risk group. We deem that drug study may bring scientific research enormous benefits and provide a novel direction.

After a comprehensive analysis of methylation-driven genes, we concluded that low-methylation, low-mutation, low-risk patients possess a better prognosis. Although most studies are based on bioinformatics, this research provided new insights for treating HCC patients and promoted the advancement of medicine.

## Discussion

The incidence and mortality of hepatocellular carcinoma are high-ranking among worldwide cancers (Siegel et al., 2021; Sung et al., 2021). HCC treatment includes non-drug and drug therapies. The former mainly include liver resection, liver transplantation, and TACE (Transcatheter arterial chemoembolization). The latter was utilized in the systematic therapy of advanced HCC, including targeted drugs (such as Lenvatinib) and monoclonal antibodies (such as nivolumab) (Chen et al., 2020). Although HCC possesses various therapies, the prognosis of patients is worse. To improve prognosis of patients, novel prognosis markers are urgently needed to explore.

DNA methylation is one of epigenetic modifications and ensures proper regulation of gene expression and stable gene silencing (Kulis and Esteller, 2010). Proper DNA methylation is necessary for normal cell development, so abnormal methylation may lead to disease, including Tumorigenesis. The study showed that DNA hypomethylation promotes Tumorigenesis through promoting chromosomal instability (Gaudet et al., 2003). On the contrary, DNA hypermethylation is the result of transcriptional suppression and decreased gene expression (Min et al., 2016). Total DNA hypomethylation plays a vital role in Tumorigenesis, which is associated with chromosomal instability (Kawano et al., 2014).

The immune cells within the tumor microenvironment (TME) play a vital role in Tumorigenesis, including tumor-antagonizing or tumor-promoting functions (Lei et al., 2020). The components of TME are closely associated with immune checkpoint blockade (ICB); ICB reactivates the effective antitumoral immune response through taking advantage of immune cell infiltration (Petitprez et al., 2020). Nowadays, partly targeting TME has been approved in solid cancer treatment (Jiang et al., 2020). Thus, studies of TME may bring enormous benefits to the treatment of HCC patients. The somatic mutations influence the development of diseases, and induction of somatic mutations promotes Tumorigenesis (Dalgliesh and Futreal, 2007; Frank, 2014).

Based on the expression data of TCGA and GSE14520, we selected 10,413 shared genes and 2850 DEGs. Next, based on the expression and DNA methylation data of TCGA, we identified the 31 methylation-driven genes. Subsequently, we perform consensus clustering analysis and establish two subtypes by taking advantage of 31 methylation-driven genes. And we found that two subtypes are featured by significant survival outcomes, immune features, and mutation status in HCC. And the establishment of HCC subtypes can correctly predict the clinical outcomes, immune features, and mutation status. Of course, Huang et al. (2020) constructed four subtypes and analyzed the correlation between different subtypes and survival, immune checkpoints, metabolism signatures, clinical feature, etc. However, the study is lack of external validation. In our study, TCGA cohort was deemed as training cohort and GSE14520 was regarded as the testing cohort. Although the consensus clustering is too rough to guide immunotherapy, the methylation-driven gene subtypes may provide a promising guideline for HCC clinical treatment.

Moreover, in order to construct a prognostic model with as few genes as possible and high accuracy, the 31 methylation-driven genes were used to perform the survival analysis to select the survival-related genes. Immediately, a panel of 5 methylation-driven genes was built by the LAASO regression model (Garcia et al., 2010; Engebretsen and Bohlin, 2019), and the patients were divided into high and low-risk groups. The model was established with TCGA as the training cohort and GSE14520 as the validation cohort. Previous study of Luo et al. (2020) built a 10-gene prognostic risk score model to predict the prognosis of HCC patients and this model showed the prediction accuracy. However, their model lacks external validation and immune associated analysis. Thus, we systematically analyzed differences in survival, immune cell infiltration, somatic mutation status, underlying mechanisms, and drug sensitivity. We deem that risk score is closely associated with OS and clinical features (gender, grade, stage, and T), and risk score serve as an independent prognosis factor of HCC patients. In addition, the risk score is closely related to immune cells based on TIMER, CIBERSORT, CIBERSORT-ABS, QUANTISEQ, MCP-counter, XCELL, and EPIC algorithms. The results of CIBORSORT algorithm showed that risk score is positively correlated with M0 macrophages and negatively correlated with naïve B cells, M1 macrophages, monocytes, resting memory CD4 T-cells, and CD 8 T-cells. Plus, TIDE was used to evaluate the clinical efficacy of immunotherapy in HCC patients. Results showed that high-risk group possesses a lower TIDE level than low-risk group, which means that high-risk group was more likely to benefit from immunotherapy. Based on the prognosis model, we perform the tumor mutation analysis. The difference of tumor mutation burden (TMB) exists in high-risk and low-risk groups. The survival analysis showed that high TMB and high-risk groups are closely related to a worse prognosis. Furthermore, to explore the underlying mechanism, we perform the GSEA analysis. We found that risk-high group is correlated with cell cycle, ECM receptor interaction, immune, etc. The ECM receptor interaction plays a significant role in tumor shedding, movement, adhesion, and hyperplasia (Bao et al., 2019). The risk-low group is closely correlated with metabolisms. OncoPredict (Maeser et al., 2021) method was utilized to perform the drug sensitivity analysis, and various drugs were explored in our study. Finally, 5 methylation-driven genes of this model were used to perform survival analysis based on the expression and DNA methylation level. And RT-PCR was utilized to test the expression of 5 genes. We found that 5 methylation-driven genes were closely associated with the survival of HCC patients, and DNA methylation levels of *CYBYR*, *CYP2C9*, and *LAMB1* were closely associated with survival of HCC patients.

*CABYR* is produced by alternative splicing, has high expression in most tumors, including HCC, and is closely correlated with a worse prognosis in HCC patients (Yu et al., 2020). The knockdown of *CABYR* chemosensitivity through inactivating *AKT* pathway in non-small cell lung cancer cells (Qian et al., 2014). *CYP2C9* is essential in drug metabolism and exogenous carcinogens in various tumors. *CYP2C9* over-expression decreases the migration and invasion of ESCC (Jiang et al., 2021). Meanwhile, *CYP2C9* is also down-regulated in HCC (Yu et al., 2015). *GNA14* is down-expressed and inhabits HCC progression through *MAPK*/*JNK* and *PI3K*/*AKT* signaling pathways (Xu et al., 2021). In addition, hypermethylation of *GNA14* promoter is upregulated in HCC (Song et al., 2021). *GNG4* is high-expressed and closely related to poor prognosis in colorectal cancer (Liang et al., 2021). *GNG4* was also explored as a downstream target of *PSMC2* in gallbladder cancer (GBC) (Zhu et al., 2021). In addition, *GNG4* promotes the progression of lung cancer (Zhou et al., 2022). However, rarely studies of *GNG4* were discovered in HCC. *LAMB1* is upregulated in gastric cancer and promotes tumor development through the ERK/c-Jun axis (Lee et al., 2021). Plus, *LAMB1* serves as a target of miR-124-5p in glioblastoma multiforme (GBM) (Chen et al., 2014). Of course, we only observed a few studies in HCC. *LAMB1* sever as a target of *DDX24* in HCC. The previous studies found that the 5 methylation-driven genes possess more excellent prospects and the tumorigenesis mechanism is unclear in HCC.

Our study analyzed the model’s predictive performance based on survival, clinical, molecular mechanism, immune landscape, tumor mutation status, and immunotherapy sensitivity. However, we also found some limitations in our study. Firstly, most studies are based on public databases, and there is an urgent need to perform more clinical validation. Secondly, most of study are based on bioinformatics analysis. This model needs further confirmation from basic experiments. Therefore, future studies will further focus on the five methylation-driven genes.

In conclusion, our study established and validated a prognostic model for HCC based on the methylation-driven genes, which were utilized to effectively predict the prognosis of HCC patients. Although our research possesses a few limitations, this study will still bring our novel insights and guide scientific progress.

## Conclusion

In summary, this study developed a novel risk model of five methylation-driven genes based on the comprehensive bioinformatics analysis, which accurately predicts survival of HCC patients and reflects the immune and mutation features of HCC. This study provides novel insights for immunotherapy of HCC patients and promotes medical progress.

## Data Availability

The original contributions presented in the study are included in the article/Supplementary Materials, further inquiries can be directed to the corresponding authors.

## Supplementary material

SUPPLEMENTARY FIGURE S1 RT-PCR was utilized to test the expression of five DNA methylation-associated genes.

## Abbreviations

*CABYR*, Calcium-binding tyrosine phosphorylation-regulated protein; CIBERSORT, the Cell Type Identification by Estimating Relative Subsets of RNA Transcripts; *CYP2C9*, Cytochrome P450 2C9; *DDX24*, DEAD-box helicase 24; DMEM, Dulbecco’s modified eagle medium; ESCC, Esophageal squamous cell carcinoma; ESTIMATE Estimation of STromal and Immune cells in MAlignant Tumor tissues; FBS, Fetal bovine serum; GEO, Gene Expression Omnibus; TCGA, Gene Expression Omnibus; TMB, Tumor Mutation Burden; *GNA14*, G Protein Subunit Alpha 14; *GNG4*, G protein subunit gamma 4; HCC, Hepatocellular Carcinoma; *LAMB1*, Laminin subunit beta 1; LIHC, Liver Hepatocellular Carcinoma; GSEA, Gene Set Enrichment Analysis; TIDE, Tumor Immune Dysfunction and Exclusion; TPM, transcripts per million.
