# Supplementary figures and images for: Identification and analysis of DNA methylation-driven signatures for prognostic and immune microenvironments evaluation in hepatocellular carcinoma
Source: Front Genet. 2022 Oct 10;13:1022078. doi: 10.3389/fgene.2022.1022078 (PMC9589435; doi:10.3389/fgene.2022.1022078)

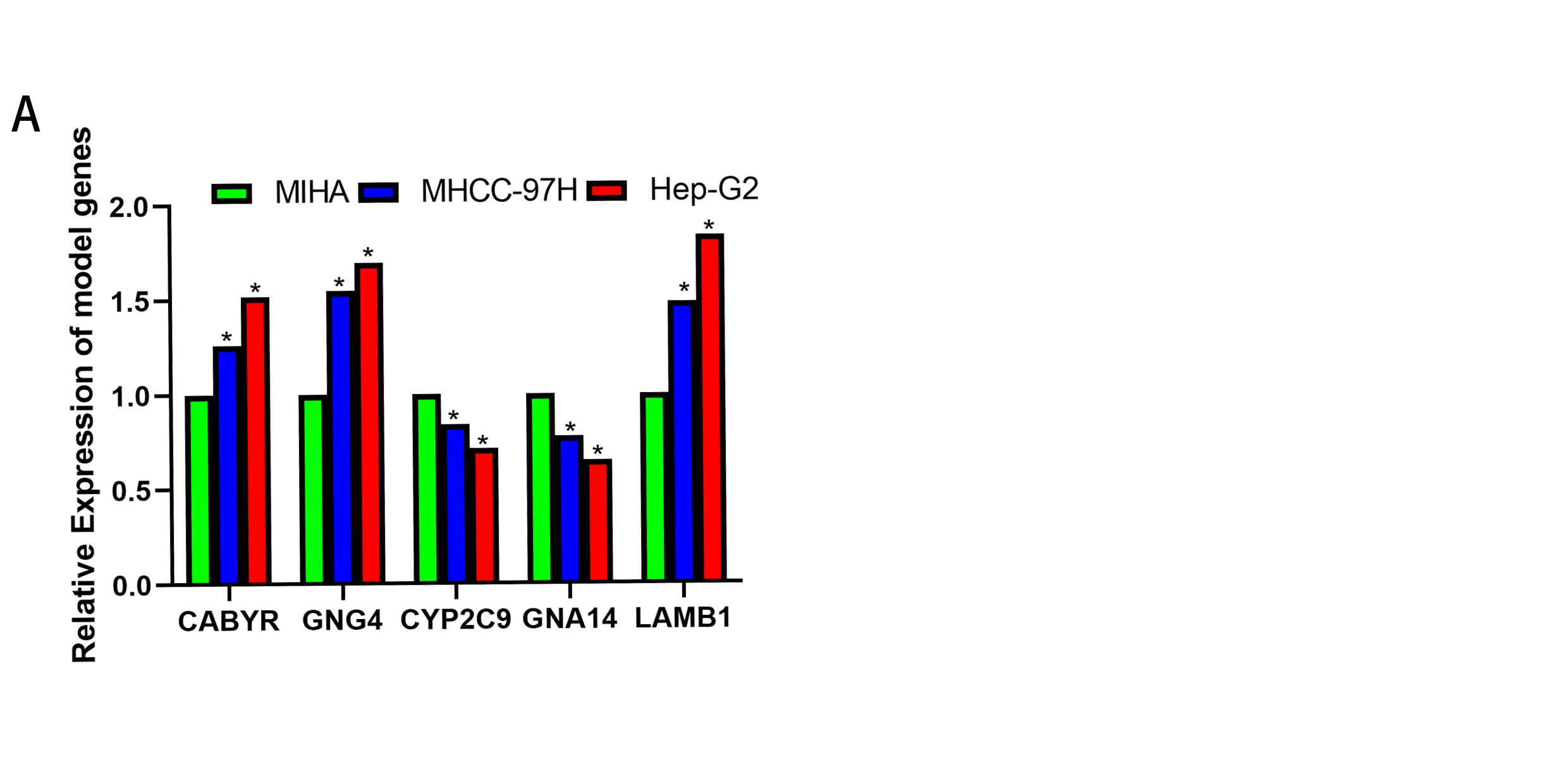

Supplement: Supplementary file 3 [file Image1.TIF]
